# Appetite and ghrelin levels in iron deficiency anemia and the effect of parenteral iron therapy: A longitudinal study

**DOI:** 10.1371/journal.pone.0234209

**Published:** 2020-06-04

**Authors:** Hanin Ghrayeb, Mazen Elias, Jeries Nashashibi, Awni Youssef, Mari Manal, Liala Mahagna, Masalha Refaat, Naama Schwartz, Adi Elias

**Affiliations:** 1 Department of Internal Medicine C, Emek Medical Center, Afula, Israel; 2 Rapaport Faculty of Medicine, Technion Institute of Technology, Haifa, Israel; 3 Department of Internal Medicine D, Rambam Health Care Campus, Haifa, Israel; 4 Bar-Ilan University Faculty of Medicine, Azrieli Faculty of medicine, Safed, Israel; 5 Nazareth Tower Out Patients Clinic Ambulatory, Clalit, Nazareth, Israel; 6 Endocrine laboratory, HaEmek Medical Center, Afula, Israel; 7 Clinical Research Unit, HaEmek Medical Center, Afula, Israel; 8 Department of Internal Medicine B, Rambam Health Care Campus, Haifa, Israel; Sahlgrenska University Hospital, SWEDEN

## Abstract

**Background:**

Iron deficiency anemia (IDA) is associated with decreased appetite. The ghrelin hormone is one of the major regulators of appetite.

**Objectives:**

To evaluate appetite and ghrelin levels in patients with IDA, and to investigate the change in appetite and ghrelin following intravenous iron therapy.

**Methods:**

A total of 56 IDA patients and 51 controls were included in the study. Both appetite and ghrelin were assessed at baseline and following intravenous iron therapy. These were assessed at corresponding time intervals in the control group. Appetite was assessed by the SNAQ score (Simplified Nutritional Appetite Questionnaire) and fasting ghrelin levels were assessed by acylated ghrelin (AG), unacylated ghrelin (UAG) and their respective ratio AG/UAG.

**Results:**

IDA patients had significantly lower SNAQ scores, yet higher AG levels and higher AG/UAG ratios compared to healthy controls; the mean SNAQ scores were 12.56 ± 3.45 and 16.1 ± 2, respectively (P<0.01); the median AG levels were 57.5 pg/ml and 43 pg/ml respectively (P = 0.007); and the median AG/UAG ratios were 0.48 and 0.25 respectively (P = 0.04). On multivariate linear regression analysis, IDA remained independently associated with decreased SNAQ score (β = -0.524, P<0.001) and increased acylated ghrelin (β = 0.289, P = 0.013). After IDA was treated, SNAQ scores increased significantly by a mean of 2 points. AG and AG/UAG ratios decreased significantly by a mean of -18.44 pg/ml and -0.2 respectively. The control group showed no significant change in SNAQ scores or ghrelin at corresponding time intervals.

**Conclusions:**

IDA patients have a reduced appetite and paradoxically elevated ghrelin hormone activity compared to healthy controls. Treating IDA enhances appetite and lowers ghrelin levels. Future studies are needed to explore the mechanism of this paradoxical ghrelin activity.

## Introduction

The prevalence of anemia in 2010 was estimated to be about one third of the word global population [1]. Iron deficiency anemia (IDA) is the leading cause of anemia in both developed and developing countries [2,3]. It is caused by increased body requirements as in pregnant women or thriving children, or in pathologic conditions, such as reduced iron intake, chronic blood loss, or gastrointestinal malabsorption [4]. Oral iron treatment is considered the first choice, with the exception of heavy bleeding or malabsorptive conditions like inflammatory bowel disease or gastric bypass. Oral iron therapy increases the incidence of gastrointestinal adverse events, such as abdominal pain and impaired appetite, which lead to low patient adherence [5]. Patients with low adherence or those who cannot tolerate oral therapy are eventually treated with intravenous iron therapy, which is highly efficacious and has a low incidence of serious adverse events [6].

Poor appetite is highly prevalent among IDA patients [7], which may also be the explanation for poor adherence to oral iron therapy. Appetite is mediated by the central nervous system via neuropeptides that regulate energy homeostasis. In addition, long-term nutritional status is mediated by insulin and by hormones synthesized by adipose tissue. Circulating gut hormones control short-term appetite [8].

Ghrelin is a peptide produced in the gastrointestinal tract; this hormone activates the ghrelin receptors expressed in various regions of the brain. Ghrelin stimulates pituitary release of the growth hormone and is involved in the hypothalamic regulation of energy homeostasis [9]. Scientific evidence links ghrelin to the overall control of energy, especially in limited energy conditions, in which this hormone demonstrates a pivotal role in energy homeostasis [10]. There are two circulating forms of ghrelin; acylated ghrelin (AG) and unacylated ghrelin (UAG). AG is considered the metabolically active fraction of the hormone that regulates appetite [11]. Ghrelin O-acyl transferase (GOAT) was recently discovered as the enzyme responsible for the hormone acylation and activation [12,13]. UAG has long been considered as the degradation product of ghrelin without a biological activity, however, recent evidence suggests it behaves like a separate hormone and might be a functional inhibitor of ghrelin [14]. Furthermore, according to some studies higher AG/UAG ratios are linked to obesity and hyperphagia [15,16].

Previous studies in pediatric populations have demonstrated lower levels of ghrelin in IDA patients, compared to healthy children, and a positive correlation between iron stores depletion and ghrelin levels, suggesting that ghrelin mediates the poor appetite in IDA [17,18]. Furthermore, children with IDA treated with oral iron therapy have demonstrated an increase in appetite and in ghrelin levels [19]. A more recent study, however, has shown a negative correlation between ghrelin levels and iron levels [20].

The aim of this study was to compare appetite and ghrelin levels in patients with IDA compared to a healthy control group, and to examine the change in appetite and ghrelin levels in patients being treated for IDA with intravenous iron. Appetite was assessed using the simplified nutrition assessment questionnaire (SNAQ) [21], while ghrelin levels were assessed by acylated ghrelin (AG) and the ratio between acylated and unacylated ghrelin (UAG), which reflects the hormone physiological activity [22,23].

## Material and methods

The study was conducted in two medical centers, HaEmek Medical Center and Nazareth Towers’ outpatient clinics, in the northeastern part of Israel. The study was approved by the HaEmek Medical Center Helsinki Institutional Review Board and Ethics Committee (ID: EMC-15-155). All participants gave written informed consent prior to the study. The protocol was registered at Clinicaltrials.gov (ID: NCT02734641).

### Study population

Individuals eligible for enrollment in the study were adults diagnosed with IDA and scheduled for intravenous iron replacement therapy. IDA was defined as hemoglobin lower than 13 g/dl in males or 12 g/dl in females and ferritin levels lower than 15 ng/ml.

Inclusion criteria were age older than 18 years, IDA unresponsive to oral iron therapy, at least four-hour fasting before taking the samples. Exclusion criteria were current or past malignancy, chronic kidney disease, acute or chronic inflammatory disease, pregnancy, functional endocrine disorders, corticosteroids treatment, anti-depressants and anti-psychotic drugs. Non-compliant patients who started intravenous iron therapy but missed their following treatment sessions were excluded from the final analysis (4–10 sessions were required to complete the treatment). In addition, a group of healthy individuals were included as a control group.

A total of 60 patients with iron deficiency anemia (IDA) were enrolled in the study group, of them 2 were excluded due to faulty blood samples and 2 were lost to follow-up. The remaining 56 patients were included in the final analysis along with 51 healthy participants in the control group, all participants in the control group remained healthy during the study period. The age range of the IDA group was (18–98) years versus (19–61) in the control group. Diagnosis of heart failure, chronic obstructive pulmonary disease and cirrhosis diagnosis were collected, there were no cases in both groups.

### Study procedure and data collection

Participants were interviewed with a detailed questionnaire that included demographic information (age, gender, smoking habits, comorbid conditions), a SNAQ appetite questionnaire, weight and height as measured at the time of enrollment, and body mass index (BMI) (weight [kg]/height^2^ [cm]). Blood samples for fasting ghrelin were collected at the time of enrollment and after completion of the once weekly, 4- to 10-week-long intravenous iron therapy in the IDA group, and a prespecified 6 weeks interval in the control group.

At the time of enrollment blood were collected with the use of a butterfly needle with tubing of 21-gauge; 5 blood samples were taken: one ethylenediaminetetraacetic acid tube (EDTA) in order to assess complete blood count, two samples for serum separating tubes for assessing basic metabolic panel and iron studies, and two samples of EDTA and phenylmethylsulfonyl fluoride (PMSF) protease inhibitor for assessing fasting ghrelin levels. Estimated glomerular filtration rate (eGFR) was calculated using the Cockcroft-Gault formula.

All participants were treated with intravenous iron sucrose. Each participant received a total dose of elemental iron in milligrams according to the hemoglobin iron deficit which is equal to body weight (kg) × (14 –Hemoglobin) (g/dL) × 2.145 + iron stores deficit which is equal to 500 (mg) [24]. This was given in 100- or 200-milligram doses each week. Once intravenous iron therapy was completed, blood was drawn in the same manner as in the last session to assess change in blood count, iron studies, and ghrelin levels. Appetite and ghrelin levels were assessed after a prespecified, 6-week interval in the control group.

Complete blood counts and basic metabolic panels were analyzed respectively by the ADVIA 2120i hematology system (Siemens Healthcare, Erlangen, Germany) and the AU5800 series chemistry analyzer (Beckman Coulter, Brea, Ca, USA).

### SNAQ- Simplified Nutritional Appetite Questionnaire

Appetite was measured with the Simplified Nutritional Appetite Questionnaire. The SNAQ includes four questions based on a numerical scale of 1 to 5.

The total SNAQ score ranged from 4 to 20. Those with SNAQ scores < 14 were reported to be at significant risk of weight loss > 5% within 6 months. The questionnaire was translated into Hebrew and Arabic by professional translators and was available to patients at the time of enrolment [21,25].

### Acylated and unacylated ghrelin

Two samples of 6 mL of blood were collected in tubes containing EDTA, a 0.05 mL 1N HCL and a 0.01 mL PMSF per 1 mL of plasma were added to the samples to prevent the degradation of acylated ghrelin.

They were centrifuged for 15 minutes at 3500 RPM at 4°C within one hour of blood collection and stored at -80°C for later analysis. Acylated and unacylated ghrelin plasma concentration were determined with the use of a sandwich-ELISA (Enzyme-linked Immunosorbent Assay) Kit, from SPI-BIO, Bertin Technologies (Montigny-le-Bretonneux, France).

### Sample size

A total of 100 participants, 50 for the IDA group and 50 for the control group, are needed in order to identify 20 pg/ml difference in acylated ghrelin levels, with expected standard deviation of 35 pg/ml and power of 80%.

### Statistical analyses

Normal distribution was assessed by visual inspection of histograms with normal curves and by means of the Shapiro-Wilk test. Categorical variables were presented with frequency and percent and continuous variables were presented with mean ± standard deviation, and median [interquartile range] in case of non-normality.

Continuous variables between the study and the control group were compared with the unpaired Student t-test and the Mann-Whitney test in case of non-normality. For each participant, the change (i.e. difference) in the SNAQ questionnaire, laboratory test results (such as hemoglobin, iron stores) and serum ghrelin levels, before and after intravenous iron treatment, was calculated.

The paired t-test (or Wilcoxon signed rank test) was used to explore significant change before and after intravenous iron treatment. The association between the continuous variables was examined by means of the Pearson or the Spearman correlation.

The associations between categorical or continuous variables and SNAQ scores and ghrelin levels were examined with multivariate general linear model (GLM). Continuous variables with non-normal distribution after logarithmic transformation. The statistical analyses were performed by IBM SPSS Statistics 21.0. Statistical significance was *P* < 0.05.

## Results

### Descriptive statistics

The baseline characteristics of the study population and the control group are summarized in Table 1. The mean age of the IDA group was 40.6 ± 11.7 years versus 37.6 ± 10 in the control group, without a significant difference between the two groups (*P* = 0.18). The mean BMI was 27.22 ± 5.27 kg/m^2^ in the IDA group versus 25 ± 3.47 in the control group (*P* = 0.018). Fifty-two (92.9%) patients were females in the IDA group versus (54.9%) in the control group (*P* < 0.01). The mean hemoglobin was 8.64 ± 1.17 gr/dL in the IDA group versus 13.89 ± 1.41 in the control group (*P* < 0.01). Hemoglobin and iron stores (iron, ferritin and transferrin) were significantly lower in the IDA group (*P* < 0.01).

**Table 1:** Baseline demographic characteristics, 56 participants with iron-deficiency anemia and 51 healthy participants.

	IDA (N = 56)	Control group (N = 51)	*P* values
Age	40.6 ± 11.7	37.6 ± 10	NS
Gender (Female)	52 (92.9%)	28 (54.9%)	<0.0001
BMI	27.22 ± 5.27	25 ± 3.47	0.017
Prior hospitalization	5 (8.9%)	0	NS
IHD	1 (1.8%)	0	NS
Cerebrovascular disease	1 (1.8%)	0	NS
Diabetes mellitus	5 (8.9%)	0	NS
Hypertension	5 (8.9%)	0	NS
Smoking	5 (8.9%)	6 (11.7%)	NS
eGFR (mL/min/1.73 m^2^)	142 ± 52	124 ± 33	0.042
Urea (mg/dL)	23.7 ± 8.23	30.3922 ± 10.42	<0.0001
Sodium (mEq/L)	138.64 ± 1.782	137.84 ± 1.826	0.029
Hemoglobin (gr/dL)	8.64 ± 1.17	13.89 ± 1.41	<0.0001
Platelets (10^3^/μL)	319 ± 106	251 ± 56	<0.0001
WBC (10^3^/mL)	6.7 ± 2.76	7 ± 2	NS
RDW (%)	17.39 ± 1.17	13.89 ± 1.41	<0.0001
Iron (μg/dL)	20.4 ± 22.29	74.3 ± 28.5	<0.0001
Ferritin (ng/dL)	4.753 ± 4.3	82.3 ± 69.3	<0.0001
Transferrin (mg/dL)	351.7 ± 32.5	278.6 ± 38.216	<0.0001

IDA, iron deficiency anemia; BMI, body mass index; IHD, ischemic heart disease; RDW, red blood cell distribution width; eGFR, estimated glomerular filtration rate; NS, non-significant, *P* > 0.05,https://en.wikipedia.org/wiki/Red_blood_cell_distribution_width Continuous variables are summarized with mean ± SD, and categorial variables with numbers and percentages.

### Appetite and ghrelin levels according to presence or absence of iron deficiency anemia

Table 2 compares the SNAQ scores and ghrelin serum levels between IDA study cohort and control group. All questionnaire scores (total SNAQ and each individual question) were significantly lower in the IDA group compared to the control group. Conversely acylated ghrelin and AG/UAG ratio were significantly higher in IDA patients compared to healthy participants. There was no significant difference in unacylated ghrelin levels between the two groups (*P* > 0.05).

**Table 2:** Comparison of appetite questionnaire and ghrelin levels between patients with iron deficiency anemia and healthy participants.

	IDA (N = 53)	Control group (N = 51)	*P* values
SNAQ (points)	12.56 ± 3.45	16.1 ± 2	<0.0001
Question 1	3.21 ± 1.16	4.27 ± 0.85	<0.0001
Question 2	2.4 ± 1.3	3.67 ± 0.816	<0.0001
Question 3	3.89 ± 1.15	4.39 ± 0.72	0.009
Question 4	3.08 ± 0.99	3.84 ± 0.85	<0.0001
Unacylated ghrelin (pg/ml) †	132 [81.1–205]	165 [93.5–231.5]	NS
Acylated ghrelin (pg/ml) †	57.5 [47–77.5]	43 [24.7–72.12]	0.007
AG/UAG ratio†	0.487 [0.25–0.777]	0.25 [0.132–0.473]	0.04

SNAQ, simplified nutrition assessment questionnaire; Continuous variables are displayed as mean ± standard deviation

† Median [interquartile range] are displayed for non-normally distributed variables, NS, non-significant, *P* > 0.05.

As shown in Table 3 on multivariable linear regression analysis that accounted for relevant potential confounders, IDA remained independently associated with decreased SNAQ score with β = -0.524, and increased acylated ghrelin levels and AG/UAG ratios, with β = 0.289 and β = 0.421 respectively.

**Table 3:** Association between iron deficiency anemia (binary variable) and SNAQ score and ghrelin levels (N = 104).

Model adjustments	SNAQ		AUG †		AG †		AG/UA †	
	β	*P*	β	*P*	β	*P*	β	*P*
Crude	-0.537	<0.001	-0.142	0.145	0.200	0.04	0.267	<0.01
Gender	-0.537	<0.001	-0.189	0.08	0.200	0.04	0.275	0.05
Age, gender, BMI, smoking	-0.489	<0.001	-0.198	0.080	0.305	0.007	0.395	<0.001
*Multivariable model	-0.524	<0.0001	-0.237	0.466	0.289	0.013	0.421	<0.0001

β, the standardized regression coefficients are shown; BMI (body mass index); SNAQ, simplified nutrition assessment questionnaire; * adjustment for age, gender, BMI, smoking status, diabetes mellitus, hypertension, eGFR, prior hospitalization

† Variables logarithmically transformed.

In order to assess the effect of gender differences on the study results, we stratified the analysis according to gender. As shown in S1 Table our results and conclusions remained robust in the female population. IDA male patients had significantly higher acylated ghrelin levels compared to the control males. Yet, they had similar SNAQ score compared to control males.

### Correlation between continuous variables and SNAQ scores and ghrelin levels

BMI had a statistically significant, negative correlation with acylated ghrelin; the correlation coefficient was 0.334. Hemoglobin also had a significant, positive correlation with iron, ferritin, and acylated ghrelin; the correlation coefficients were 0.472, 0.464, and 0.271 respectively. Ferritin also had a significant positive correlation with iron and SNAQ score; the correlation coefficients were 0.395 and 0.363 respectively. No significant correlation was found between the SNAQ score and ghrelin levels (Table 4). Comparison of the ghrelin levels between two SNAQ score categories; SNAQ>14 versus SNAQ ≥14 showed no significant difference (*P* > 0.05), the mean AG were 58.13 ± 27.11 versus 72.66 ± 43 respectively.

**Table 4:** Spearman/Pearson correlation coefficients for the relationship between continuous variables in patients with iron deficiency anemia before treatment (N = 53).

	Age	BMI	Hemoglobin	Iron	Ferritin	Transferrin	UA-Ghrelin	A-Ghrelin	SNAQ
Age	1	0.290*	-0.241	-0.056	0.236	-0.274	0.209	-0.237	0.047
BMI	0.290*	1	-0.313*	-0.087	0.186	-0.139	-0.023	-0.334*	-0.163
Hemoglobin	-0.241	-0.313*	1	0.472**	0.464**	-0.021	0.030	0.271*	0.208
Iron	-0.056	-0.087	0.472**	1	0.395**	0.132	0.160	0.176	0.211
Ferritin	0.236	0.186	0.464**	0.395**	1	-0.123	0.177	0.137	0.363**
Transferrin	-0.274	-0.139	-0.021	0.132	-0.123	1	0.185	0.051	-0.117
SNAQ	0.047	-0.163	0.208	0.211	0.363**	-0.117	0.196	0.176	1

BMI, body mass index; UAG, unacylated ghrelin (pg/ml); AG, acylated ghrelin (pg/ml); SNAQ, simplified nutrition assessment questionnaire

*; *P* < 0.05

**; *P* < 0.01.

### Change in SNAQ and ghrelin levels after intravenous iron therapy

**Fig 1:**
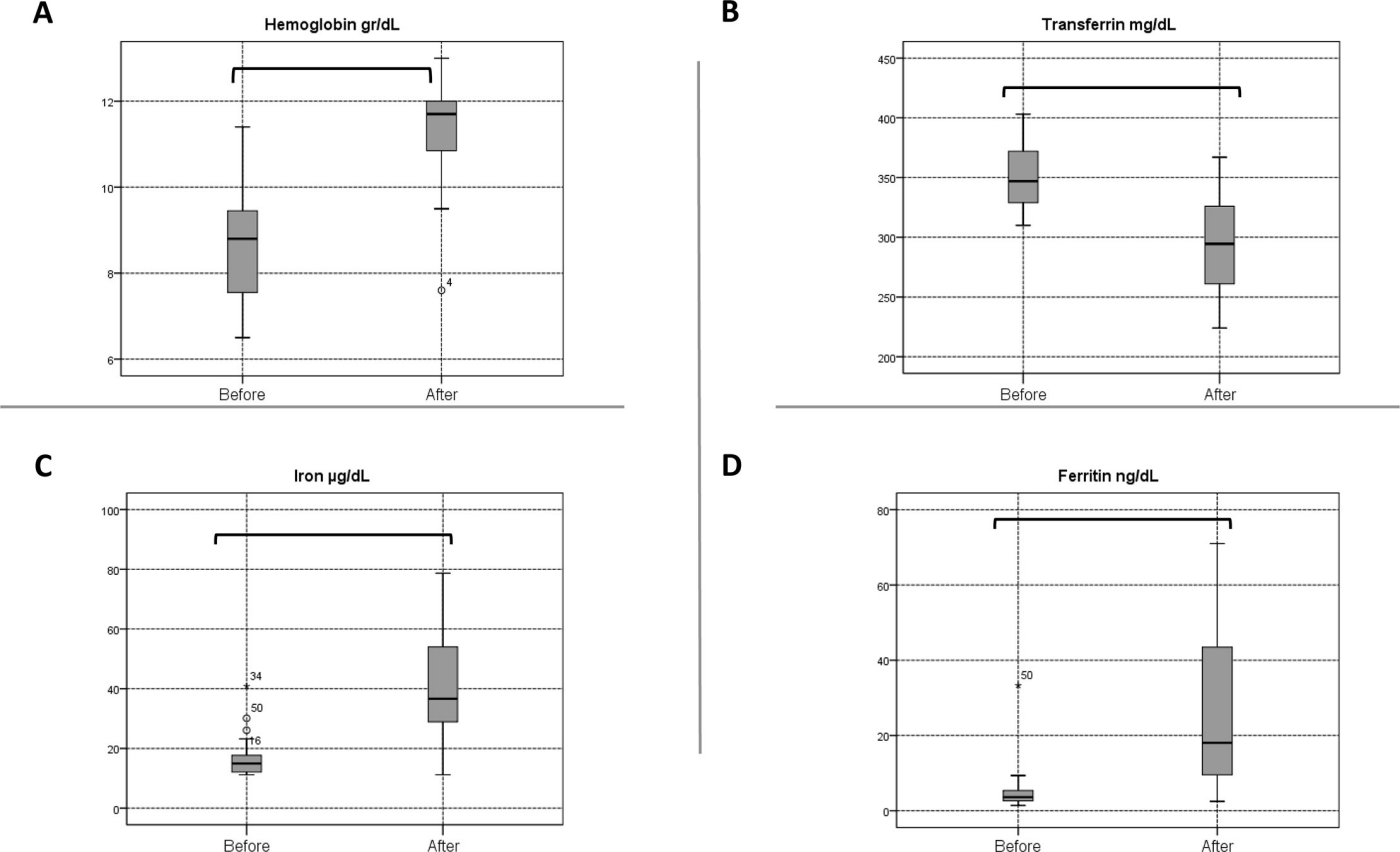
Box plots for hemoglobin and iron studies at baseline and after treatment. The horizontal line inside each box indicates the median, while the bottom and top of each box indicate the 25^th^ and 75^th^ percentile, respectively. The bars indicate the upper adjacent value (75th percentile plus 1.5 time the interquartile range) and the lower adjacent value (25th percentile minus 1.5 times the interquartile range). Empty circles indicate outliers, and brackets indicate statistical significance (*P* < 0.05).

**Fig 2:**
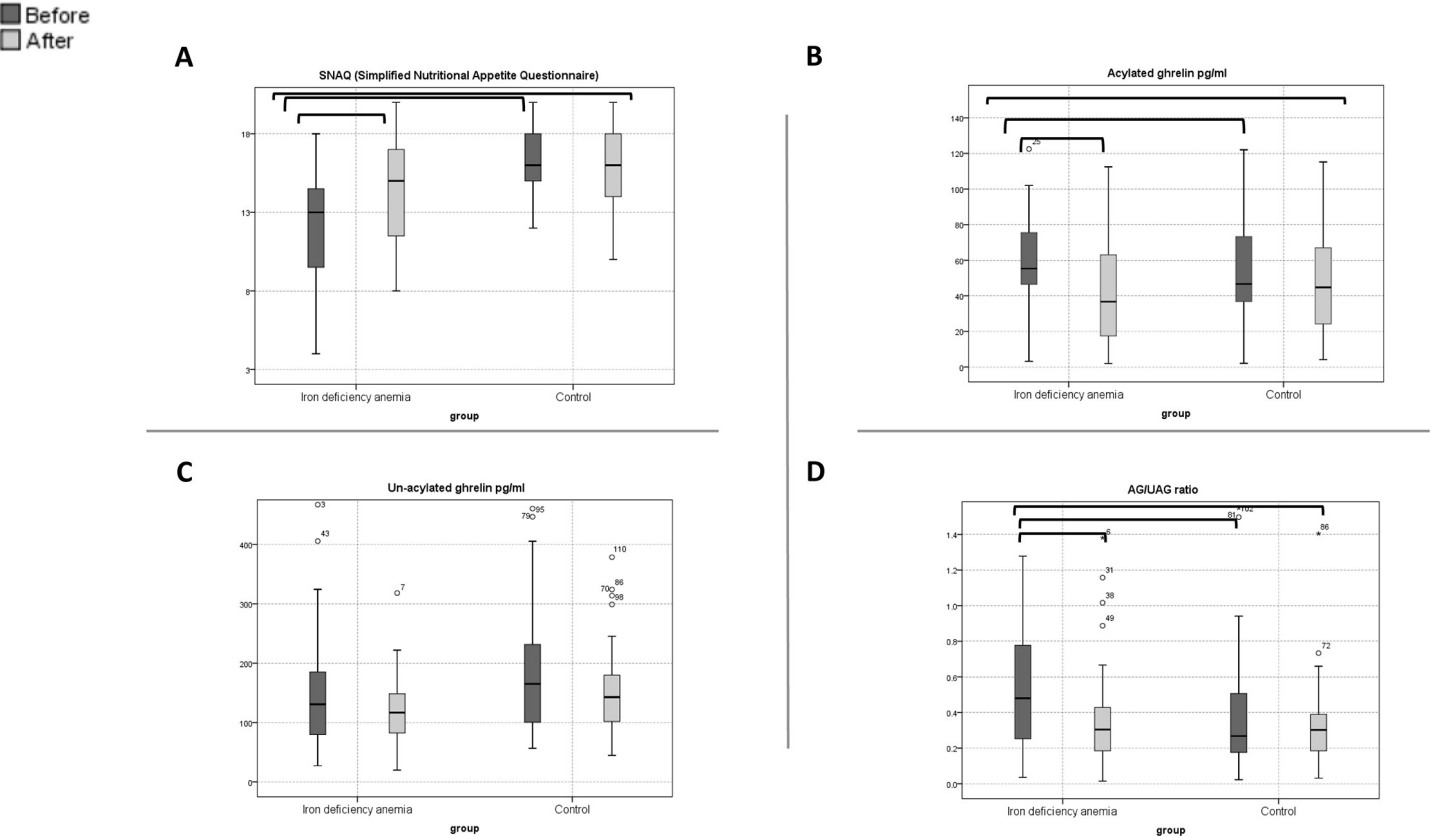
Box plots for SNAQ score, ghrelin levels according to groups, IDA versus healthy control at baseline and after treatment. The horizontal line inside each box indicates the median, while the bottom and top of each box indicate the 25^th^ and 75^th^ percentile, respectively. The bars indicate the upper adjacent value (75th percentile plus 1.5 time the interquartile range) and the lower adjacent value (25th percentile minus 1.5 times the interquartile range). Empty circles indicate outliers, and brackets indicate statistical significance (*P* < 0.05).

Table 5 summarizes variables at baseline and after intravenous iron therapy was completed after a mean interval of 7.6 ± 1.98 weeks. The mean total dose of iron sucrose was 764 ± 198 milligrams; the mean increase in hemoglobin levels was 2.8 g/dL (*P* < 0.01). The hemoglobin increased above 2 g/dL in 73.2% and above 1 g/dL in 94.4% following iron therapy (Fig 1). The mean change in iron, transferrin, and ferritin levels were 26.5, -57 and 31.5 respectively (*P* < 0.01). The mean change in SNAQ score was 2 (*P* = 0.013). Acylated, unacylated ghrelin, AG/UAG ratio decreased significantly after completion of iron therapy (Fig 2); the mean change was -47.58, -18.44 and -0.2 (*P* = 0.045, *P* = 0.0001 and 0.003) respectively (Table 5) and (Fig 1).

**Table 5:** Comparison of hemoglobin, iron studies, ghrelin and SNAQ at baseline and after treatment.

	*IDA (N = 45)*				Control (N = 44)			
Parameters	Baseline	After treatment	Difference (CI 95%)	*P* value	Baseline	After 6 weeks	Difference (CI 95%)	P value
Hemoglobin (gr/dL)	8.5 ± 1.22	11.38 ± 1.11	2.8 (2.41 to 3.2)	<0.0001	13.89 ± 1.41	13.7 ± 1.3	-0.2 (-0.76 to 0.367	NS
Iron (μg/dL)	16.75 ± 26	43.27 ± 43	26.5 (16.17 to 36.9)	<0.0001	74.3 ± 28.5	N/A	-	-
Transferrin (mg/dL)	351 ± 26.7	294 ± 43	-57 (-77 to -36.9)	<0.0001	278 ± 38.21	N/A	-	-
Ferritin (ng/dL)	5.11 ± 5	36.7 ± 55	31.5 (11.9 to 51.2)	0.003	82.3 ± 69.3	N/A	-	-
Question 1	3.09 ± 1	3.54 ± 1.19	0.457 (-0.128 to 1)	NS	4.3 ± 0.795	4.25 ± 0.86	-0.045 (-0.28 to 0.19)	NS
Question 2	2.09 ± 1.24	2.86 ± 1.24	0.771 (0.115 to 1.42)	0.023	3.7 ± 0.79	3.5 ±0.95	-0.205 (-0.50 to 0.093)	NS
Question 3	3.86 ± 1.14	4.11 ± 0.993	0.257 (-0.245 to 0.759)	NS	4.36 ± 0.750	4.57 ± 0.661	0.205 (-0.037 to 0.44)	NS
Question 4	3.09 ± 0.95	3.86 ± 1.14	0.6 (0.174 to 1)	0.007	3.89 ± 0.868	3.70 ± 0.978	-0.182 (-0.36 to -0.005)	0.044
SNAQ (points)	12.11 ± 3.16	14.2 ± 3.39	2 (3.7 to 0.465)	0.013	16.25 ± 2	16.02 ± 2.57	-0.2272 (0.3 to -0.76)	NS
UAG (pg/ml)	168 ± 148	120 ± 54	-47.58 (-1.2 to -93.9)	0.045	193.8 ± 122.2	159.6 ± 93	- 34.2 (-2.59 to -65.8)	0.035
AG (pg/ml)	63.8 ± 37.9	45.4 ± 42.1	-18.44 (-10.97 to -25.91)	<0.0001	63.98 ± 62.8	60.46 ± 73.59	- 3.52 (8.98 to -16)	NS
AG/UAG ratio	0.563 ± 0.41	0.357 ± 0.28	-0.2 (-0.07 to -0.33)	0.003	0.39 ± 0.34	0.35 ± 0.3	-0.33 (-0.06 to -0.134)	NS

SNAQ, simplified nutrition assessment questionnaire; UAG, unacylated ghrelin; AG, acylated ghrelin; N/A, not available; NS, non-significant, *P* > 0.05; CI, Confidence Interval.

In the control group the SNAQ score and acylated ghrelin levels after 6 weeks did not differ from baseline. Yet, both question 4 and unacylated ghrelin decreased significantly; the mean change was -0.182 and -34.2 respectively (*P* < 0.05) (Table 5).

Following treatment, the SNAQ score remained significantly lower in the IDA group compared to the control group, the mean SNAQ score was 14.2 ± 3.39 and 16 ± 2.57 respectively (P = 0.006). However, there was no significant difference in the acyelated ghrelin levels between the two groups (Fig 2). Neither the change in the SNAQ nor the ghrelin parameters showed a statistically significant correlation with change of hemoglobin, ferritin, and iron levels (S2 Table).

## Discussion

According to our clinical observations and previous studies [7], patients with iron deficiency anemia suffer from decreased appetite: our study demonstrated that IDA patients have decreased SNAQ with a mean score of 12 compared to 16 in a healthy control group. This finding suggests that IDA patients are at increased risk of malnutrition [21,26]. This may partially explain the low adherence to oral iron therapy in this population, in addition to its gastrointestinal adverse effects. To our knowledge this is the first study to investigate appetite in an IDA adult population and demonstrate the increased risk for malnutrition by a validated score. Yet, the level of acylated ghrelin, a major regulator of appetite, was significantly increased in IDA patients compared to the control group (Table 2).

There was nonetheless no significant correlation between the SNAQ score and acylated ghrelin. Ghrelin levels did not differ in patients classified as high risk for malnutrition (SNAQ score < 14) compared to patients at low risk for malnutrition (SNAQ ≥ 14) in the IDA cohort. It is important to emphasize that the sample size of the study was not designed to assess the correlation between those parameters. In order to detect correlation coefficient of 0.3 with statistical power of 80%, we need at least to assess 85 patients.

As shown in Table 1, IDA patients had higher BMI compared to control group, overweight individuals are usually consider at low risk for malnutrition, yet they might be susceptible to malnutrition due to inadequate intake of iron, vitamins and other trace elements, likely as a result of poor diet quality [27]. Furthermore, our results remained statistically significant after adjustment for BMI in the multivariable model (Table 3).

Furthermore, intravenous iron replacement therapy was successful in treating IDA and increased haemoglobin levels in most patients, as mentioned above in the results. The SNAQ score was significantly elevated compared to baseline, following intravenous iron treatment, thus pointing to increased appetite and lower risk for malnutrition following this intervention. On the other hand, intravenous iron replacement significantly decreased acylated ghrelin compared to baseline levels. These changes in SNAQ scores and ghrelin levels were not demonstrated in the healthy control group, as ghrelin levels were sampled in a similar time-range interval, suggesting that time interval was not a major confounder (Table 5). The correlation between hemoglobin and ferritin change did not correlate with the change in SNAQ and ghrelin levels (S1 Table), this suggests that the change in appetite and ghrelin is independent of the change in hemoglobin and iron stores. Even though the mean SNAQ score was increased and the ghrelin levels were decreased following iron therapy, the SNAQ score remained significantly lower in the IDA group when compared to the control group, suggesting that IDA patients remain at higher risk for malnutrition compared to healthy control population (Table 5).

According to our results intravenous iron repletion increased appetite. Our observation is in line with a study from Lawless et al. on iron deficient children in Kenya, this study demonstrated improvement in appetite and growth following iron supplementation [28]. Another study on iron deficient children from Stoltzfus et al. demonstrated improvement in appetite and iron stores following low dose iron supplementation, even though iron`s effect on anemia was limited due to infection and other nutrient deficiencies [29].

Since nutrition is the only source for body iron supply [5], IDA patients who suffer from anorexia might fail to replete their iron stores whether by nutrition or by oral iron therapy. Our study demonstrated significant improvement in appetite following intravenous iron treatment. Hence, intravenous iron treatment might be a good utility to break this vicious cycle.

Our findings are incongruous with previous studies in the pediatric population [17–19], which demonstrated lower total ghrelin levels in IDA children compared to a healthy control group, and elevated ghrelin levels following oral iron therapy. There are several explanations for this discrepancy. Firstly, our study population was composed of adult versus pediatric populations, and according to previous studies acylated ghrelin levels are independently correlated with age, and a negative correlation exists between age and ghrelin levels in children [30]^,^. Secondly, in our study we measured acylated and un-acylated ghrelin levels, as well as the ratio between both fractions, as opposed to total ghrelin assays level in the aforementioned studies. Currently, acylated ghrelin levels reflect the hormone physiological activity of ghrelin more than total ghrelin levels [22,23]

Paradoxically increased levels of acylated ghrelin were demonstrated in restrictive anorexia nervosa [31–33] which might be reversible following weight gain [34], and in patients with malignancies suffering from cachexia [35]. According to previous studies, acylated ghrelin levels and the expression of the preproghrelin gene is upregulated in low energy states in order to increase both body weight and fat tissue [36]. Similarly, iron deficiency anemia could be considered a malnutritional state, and could therefore cause a compensatory elevation in acylated ghrelin in order to increase and replete iron stores.

Furthermore, according to previous studies ghrelin regulates gastric acid secretion like gastrin [37,38], and gastric acidity has a pivotal role in iron absorption [39,40]. Therefore, ghrelin might be upregulated in order to increase gastric acid secretion and enhance iron absorption in IDA patients, yet this remains only speculative until further research addresses this question.

Interestingly, using the algorithm proposed by Campillos et al. to search for iron response elements (IRE), the ghrelin O-acyl transferase (GOAT) gene appears to have nucleotide sequences that might have IRE that regulate the transcription of the GOAT [41]. However, the ghrelin gene did not have any IRE. This needs further evaluation and future research at the molecular level.

Leptin another important hormone that induces satiety and regulates energy homeostasis, this hormone secretion is regulated by adipose tissue iron [42]. According to recent study leptin was negatively regulated by iron levels in adipose tissue. Furthermore, animal models suggest that leptin hormone transcription via cAMP-responsive element binding protein activation (CREB activation) is negatively regulated by iron [43]. Therefore, IDA patients have low iron stores and high leptin levels that might induce satiety and low appetite.

Another explanation could possibly be development of central hypothalamic ghrelin resistance, which causes a disproportionate increase in ghrelin levels in several catabolic states, such as cachexia in oncologic populations in human and animals studies [44,45]. and in diet induced obesity [46–48]. Several facts might point to acquired ghrelin resistance similar to the aforementioned studies; first of all, IDA patients had decreased appetite and paradoxically high activity of ghrelin that could be due to high counterregulatory action of leptin as mentioned before [43]. Secondly, there was no correlation between ghrelin activity and appetite in the IDA group. Moreover, the reversibility of both appetite and ghrelin following iron treatment support this assumption. Yet this remains only speculative.

This paradoxical association between ghrelin and its biological activity on appetite points to a major hormonal dysregulation in IDA patients. As mentioned above this association might be explained by compensatory ghrelin elevation or acquired ghrelin resistance. Additional studies are needed to explore those hypotheses.

### Study limitations

Unfortunately, our study suffered from several limitations. One of the study limitations was age, BMI, gender, and comorbidity differences between IDA patients and the healthy control group. However, the association between IDA and SNAQ score and acylated ghrelin remained statistically significant after adjustment for potential and available confounders such as age, BMI, gender, smoking status, diabetes, essential hypertension, and cardiovascular diseases (Table 3).

Another possible hypothesis is that anorexia was caused by the underlying IDA etiologies, and that appetite improved following treatment of those etiologies. This assumption is less probable; since none of the study participants reported initiating new treatments during the study period. Yet, it cannot be definitely excluded.

Another limitation was the gender differences between the IDA group and the control group. However, our results remained statistically significant after adjusting for gender (Table 3). Furthermore, as shown in the subgroup analysis according to gender (S1 Table), acylated ghrelin levels remained statistically significant in both genders. However, SNAQ score were significantly lower in the female group but not in the case of the males, probably due to the small number of male participants in the IDA group. Therefore, our results regarding men should be interpreted with caution.

In addition, the time interval was shorter in the control group, however its effect should be negligible. Our results are also limited to iron deficiency in adults who failed to respond to the oral iron therapy, and must not be extrapolated to other populations, such as pediatric and oncological patients, or to pregnant women, all of which were excluded.

## Conclusion

Patients with iron deficiency anemia have decreased appetite and have an increased risk of malnutrition. Paradoxically, this group of patients has increased ghrelin levels compared to healthy participants. Intravenous iron therapy increases appetite levels, lowers the risk for malnutrition and reduces acylated ghrelin levels in iron deficiency anemia. Future studies are needed to explore the mechanism of this paradoxical ghrelin activity.

## Supporting information

S1 Table Subgroup analysis according to gender of appetite questionnaire and ghrelin levels between patients with iron deficiency anemia and healthy participants. (DOCX)

S2 Table Correlation between the change in hemoglobin, ferritin, and iron and the change in SNAQ and Ghrelin levels after the iron treatment. (DOCX)

S1 File Summary of research data. (XLSX)
