# Supplementary material for: Appetite and ghrelin levels in iron deficiency anemia and the effect of parenteral iron therapy: A longitudinal study
Source: PLoS One. 2020 Jun 4;15(6):e0234209. doi: 10.1371/journal.pone.0234209 (PMC7272047; doi:10.1371/journal.pone.0234209)
Supplement: S1 Table — (DOCX) [file pone.0234209.s001.docx]

|  | Female Population | |  | Male Population | |  |
| --- | --- | --- | --- | --- | --- | --- |
|  | IDA (N=51) | Control group (N=28) | *P* values | IDA (N=4) | Control group (N=23) | *P* values |
| Age | 39 (18-58) | 41 (19-61) | *NS* | 68.5 (64-79) | 31 (27-57) | *0.041* |
| BMI | 27.26 ± 5.39 | 24.16 ± 3.66 | 0.009 | 26.6 [23-30.47] | 26.36 [23.6-27.4] | *NS* |
| eGFR (mL/min/1.73 m^2^) | 155.6 ± 37 | 107.8 ± 27.4 | *NS* | 78.2 [65.5-137] | 140 [123-163] | *NS* |
| Hemoglobin (gr/dL) | 8.64 ± 0.19 | 12.96 ± 0.85 | <0.0001 | 8.75 ± 0.93 | 15 ± 1 | <0.0001 |
| SNAQ | 12.46 ± 3.37 | 16 ± 2.14 | <0.0001 | 15 [8.75-17.5] | 16 [15-18] | *NS* |
| Unacylated ghrelin (pg/ml) | 167.33± 147.17 | 213.450±128.46 | *NS* | 202 [144-237] | 127 [80-177] | *NS* |
| Acylated ghrelin (pg/ml) | 62.98 ± 27 | 50.1 ± 24.89 | 0.043 | 48.4 [45.7-199] | 40.5 [17-83] | 0.041 |
| AG/UAG ratio | 0.565 ± 0.367 | 0.3 ± 0.22 | 0.001 | 0.23 [0.2-1.47] | 0.32 [0.12-0.6] | *NS* |

SNAQ, short nutrition assessment questionnaire; eGFR, estimated glomerular filtration rate; Continuous variables are displayed as mean ± standard deviation, Median [interquartile range] are displayed for non-normally distributed variables, NS, non-significant, *P* > 0.05.
