# Supplementary material for: Appetite and ghrelin levels in iron deficiency anemia and the effect of parenteral iron therapy: A longitudinal study
Source: PLoS One. 2020 Jun 4;15(6):e0234209. doi: 10.1371/journal.pone.0234209 (PMC7272047; doi:10.1371/journal.pone.0234209)
Supplement: S2 Table — (DOCX) [file pone.0234209.s002.docx]

S2 Table. Correlation between the change in hemoglobin, ferritin, and iron and the change in SNAQ and Ghrelin levels after the iron treatment.

|  | ▲ Hemoglobin | | ▲ Ferritin | | ▲ Iron | |
| --- | --- | --- | --- | --- | --- | --- |
| Parameters | Correlation | *P* Value | Correlation | *P* Value | Correlation | *P* Value |
| ▲ SNAQ | 0.013 | 0.947 | -0.318 | 0.140 | -0.437 | 0.061 |
| ▼ UAG (pg/ml) | -0.112 | 0.498 | 0.190 | 0.334 | 0.208 | 0.307 |
| ▼ AG (pg/ml) | 0.098 | 0.558 | 0.151 | 0.451 | -0.004 | 0.985 |
| ▼ AG/UAG | 0.01 | 0.997 | 0.091 | 0.651 | 0.083 | 0.692 |

SNAQ, short nutrition assessment questionnaire; UAG, unacylated ghrelin; AG, acylated ghrelin; NS, non-significant, *P* > 0.05.
